# Relevance of Gender and Social Support in Self-Rated Health and Life Satisfaction in Elderly Spanish People

**DOI:** 10.3390/ijerph16152725

**Published:** 2019-07-31

**Authors:** M. Pilar Matud, M. Concepción García, Demelza Fortes

**Affiliations:** Department of Clinical Psychology, Psychobiology and Methodology, Universidad de La Laguna, 38207 La Laguna, Spain

**Keywords:** gender, social support, life satisfaction, older people, social determinants of health

## Abstract

*Background*: Gender and social support are important social determinants of health, but the relevance of such variables in older people’s health has raised less scholarly attention than in younger age groups. This study examines the relevance of gender and social support in the self-rated health and life satisfaction of elderly Spanish people. A cross-sectional study with a sample of 702 men and 754 women aged between 60 and 94 years was conducted. All participants were evaluated through questionnaires that assess gender role traits, social support, and life satisfaction. *Results*: Men scored higher than women in masculine/instrumental trait and in life satisfaction whereas women scored higher than men in feminine/expressive trait. Results from multiple regression analyses indicated that women and men presenting higher social support had better self-rated health and higher life satisfaction. High scores in masculine/instrumental trait also proved to be an important predictor of men’s and women’s high life satisfaction and of women’s better self-rated health, whereas the high feminine/expressive trait predicted better self-rated health in the men group. A high educational level was associated in the women’s group with better self-rated health and higher life satisfaction. *Conclusions*: We conclude that gender and social support are important social determinants of health among older people.

## 1. Introduction

Human health is related to social factors [1]. The socioeconomic, political, environmental and cultural factors that shape health are known as the social determinants of health, and refer to the conditions in which people are born, grow, live, work, and age [2]. Inequalities in the distribution of power, money, and resources are responsible for health dissimilarities within and among countries [2]. The social risk factors can be at the individual level—a series of attributes such as gender, race, income/wealth and educational attainment determining the position of the individual in hierarchies of power, social status and economic resources—and at the community level, which refers the circumstances people live in and which are often the product of political decisions [1]. As Marmot ([3], p. 88) asserts “People’s lives and health are shaped by the norms, values and structures of society: processes of inclusion, exclusion, vulnerability and disadvantage; the physical environment in which they live and work; and the economic and social support society and government provide”. Moreover, there is a social gradient in health, since people and communities have progressively better health, the better their socioeconomic position/conditions are [3]. Research on the social determinants of health can make out at least two distinct strands. The first one addresses the ‘health gap’ and centers on the importance of structural conditions in people’s live with reference to a range of negative health outcomes. The second strand or ‘social cure’ draws attention to the way in which personal relationships, social networks and social support feed into health outcomes [4].

### 1.1. Gender and Social Support as Social Determinants of Health

Gender, an important social determinant of health [5,6] describes “the roles, behaviours, activities, attributes and opportunities that any society considers appropriate for boys and girls, and men and women. Gender also refers to relationships between people and can reflect the distribution of power within those relationships” ([5], p. 644). Gender relations of power feature prominently among the most influential of the social determinants of health [7]. Although there is empirical evidence that women and men are similar in most psychological traits and behaviors [8], the majority of societies consider they are different and should occupy different roles; consequently, they are socialized differently according to the sex they are assigned at birth. Sex typing is the process whereby society transmutes males and females into masculine and feminine [9]. Masculinity and femininity designate the features, behaviours, and interests that society considers appropriate for each gender. Masculinity is associated with an instrumental orientation, central to which is agency, which is characterized by focusing on the self, prioritizing independence, and the achievement of personal goals. On the contrary, femininity is linked to an expressive orientation, to which communion, defined as focusing on the others, is central [10,11]. Despite the changes that have taken place in Western societies, which highlight women’s access to higher education and participation in the workforce, gender stereotypes still persist [12]. Gender stereotypes characterize men and women as complementary: women are perceived to be communal but not agentic whereas men are perceived to be agentic but not communal [13]. Such stereotypes do not only describe typical differences between women and men, as they also prescribe what and how they should behave, to the point of influencing on the way women and men define themselves and are treated by others [14].

Research on the relationships between femininity and masculinity-related constructs and health-related outcomes has identified complex relationships [15,16], although the results are influenced by the important change that the masculinity construct has undergone since the 60 s: it evolved from being viewed as a dimension to being considered socially constructed and developing scales to measure its three major and interrelated aspects, masculinity ideologies, conformity to masculine norms, and gender role conflict [17]. Although masculine norms vary by place and time [18], research on conformity to masculine norms has reported that individuals’ conformity to masculine norms is related with a range of negative psychological outcomes and reduced help seeking, although the results may vary depending on the specific dimension of the masculine norm [16]. The masculine/instrumental trait has been found to be more associated with the well-being of men and women than the feminine/expressive trait [19,20,21,22,23]; however, it has also been found that femininity is associated with optimal mental functioning [22,23,24]. It has been suggested that the strict adherence to masculine and feminine roles can limit the range of potential behaviours and choices of women and men [25], which would entail a limitation to the development of those personal characteristics which do not conform to what society considers appropriate to each gender. So, for example, women following the dictates of femininity of empathy and caring for others can prioritize the welfare of others and take care of all domestic tasks and of the care for the elderly and/or sick, thus lacking time for themselves and for activities that benefit their health (i.e., sports); while men following masculinity norms of strength and dominance can perform risky behaviors for their health such as non-help seeking, poor eating habits or alcohol abuse.

Research on the differences between women and men in health has shown complex results. Globally, the average life expectancy of men is lower than that of women [26], although women report poorer health [27] and more distress and chronic conditions than men [22,28]. But gender differences in health may vary according to other variables such as occupational grade, perceived working conditions and orientation to gender roles [29]. Furthermore, it has been found that differences in morbidity and mortality rates between women and men vary across the European Region and are changing in many countries [3].

A frequently used indicator for studying gender differences in health is self-rated health. Self-rated health is a widespread method for assessing health perceptions in populations which has been extensively employed in health research and practice as a marker of general health [30,31]. Self-rated health has proved to be a multidimensional concept that includes, in addition to the subject’s self-assessment of his/her physical health, the extent to which subjects are able to manage themselves (functional dimension), the extent to which they have adapted or their attitudes towards the disease one has (coping dimension) and how one feels (dimension of well-being) [32]. Self-rated health correlates with historical and current hospitalization and diagnosis and with future hospitalizations [33]; it has also been shown that it is an important predictor of mortality and morbidity [33,34,35]. Although several studies have found that women present worse self-rated health than men [36,37], the literature of the gender differences in self-rated health shows that such differences vary depending on other variables such as age and country of residence [30,38]. Worse self-rated health in women with respect to men has also been found in old age [37], but gender differences vary according to age group [38]. Thus, Dahlin et al. [38] found that women rated their health as poorer than men, especially among those aged between 65 and 79, yet gender differences decreased in those aged 80 to 84 years.

The importance of social connections and support for health and well-being is well-documented [39,40,41]. Research indicates that social support and social integration are protective against mortality [42], and high social support has been related to better self-rated health [43,44] and to high levels of life satisfaction around the world [44]. The benefits of social support in health and well-being have also been found in older individuals, “however the mechanism remains poorly understood” ([41], p. 1050).

### 1.2. Subjective Well-being

In 1948 the World Health Organization (WHO) defined health not merely as the absence of illness or infirmity, but in a broader sense as “a state of complete physical, mental and social well-being” ([45], p. 1); over the last decades the relevance of well-being has been confirmed. This is a complex construct which “refers to optimal psychological functioning and experience” ([46], p. 142). Well-being research distinguishes two broad traditions, the hedonistic and the eudaimonic [46]. In hedonic well-being, the search for pleasure is central, and from this perspective the focus is on subjective well-being, which includes components such as happiness, life satisfaction and positive affect [47]. Health and subjective well-being are closely related and the link could become more important at older ages [48]. Research has reported a close relationship between subjective well-being and physical and psychological health in later life; moreover, subjective well-being is a reliable predictor for mortality among middle-aged and elderly people [49]. Life satisfaction is a key indicator of subjective bell-being [50] as well as an important indicator of quality of life in social gerontology [51]. Subjective well-being is also intimately associated with health and longer survival [48,52,53,54]. In the case of the elderly it has been found that life satisfaction is related to self-rated health, sleep quality and activities of daily living [55,56], to health-related biomarker of inflammation [57] and to lower depression [56,58]. However, the studies on differences in well-being between women and men have not produced conclusive results. In general, no significant differences are found in the mean scores for subjective well-being between women and men, yet women experience positive and negative emotions more frequently and with greater intensity than men [59].

### 1.3. Aims of the Study and Hypotheses

Although in recent decades the social determinants of health have received considerable attention as a fundamental concept in the field of public health [60], the analyses of health inequalities have been fewer in the case of older people than in younger age, maybe because of the tacit assumption that illness and disability are inevitable in older age [3]. In spite of this, there is evidence that older people experience persistent health inequalities, especially determined by the socioeconomic level, the educational level and gender [3]. Thus, the main aim of the work is to analyze the relevance that two important social determinants of health, gender and social support, have on self-rated health and life satisfaction in elderly Spanish people. A second aim is to know the relevance of the sociodemographic characteristics of age and educational level in the elderly’ self-rated health and life satisfaction.

Based on previous research, it was hypothesized that men will have better self-rated health than women. The second hypothesis predicted that women and men with more educational level will present better self-rated health and greater life satisfaction. The third hypothesis predicted that women and men with higher scores on the masculine/instrumental trait will have better self-rated health and greater life satisfaction. And the fourth hypothesis predicted that women and men with higher scores on social support will report better self-rated health and greater life satisfaction.

## 2. Materials and Methods

### 2.1. Participants

The sample consisted of 702 men and 754 women aged between 60 and 94 years, who voluntarily participated in the study, and resided in eleven autonomous communities located in the different geographical areas of Spain. Their mean age (*M*) was 68 years, and the standard deviation (*SD*) of 6.52. Most of the sample (94.4%) had at least one child, being the range of the number of children between 0 and 12 (*M* = 2.51, *SD* = 1.56). Table 1 presents the main sociodemographic characteristics of women and men. As can be observed, women and men did not differ in age and number of children, but there were statistically significant differences in education level and marital status. Although there was diversity in education, most often they had only elementary studies, which occurred in 44.4% of women and 36.5% of men. Furthermore, not having completed elementary studies was more frequent in women (16.7%) than in men (14.0%). Men would more frequently have a 5-year university degree or a high school degree than women. The analysis of marital status also showed great diversity, although most often they were married or living with a partner, which was more common among men (78.3%) than among women (56.9%). Furthermore, the percentages of widowed women were higher (27.1%) than in the case of men (9.0%).

### 2.2. Measures

#### 2.2.1. Dependent Variables: Self-Rated Health and Life Satisfaction

Participants’ self-rated health was assessed by one item asking respondents to rate their overall health at the present time on a five-point scale. The possible choices were: “very good”, “good”, “moderate”, “bad”, “very bad”. Scores were assigned from 0 (for “very bad”) to 4 (for “very good”).

The Satisfaction with Life Scale (SWLS) [61] was used to assess life satisfaction. The SWLS is made up of 5 items rated on a 7-point Likert scale ranging from 1 (strongly disagree) to 7 (strongly agree). The minimum possible value was 5 and the maximum was 35, and high scores indicated a greater life satisfaction. All items were translated into Spanish and back translated into English by the research team plus two bilingual persons. For the current sample, the Cronbach’s alpha coefficient was 0.86 in the men’s group and 0.87 in the women’s group.

#### 2.2.2. Independent Variables: Masculine/Instrumental Trait, Feminine/Expressive Trait, Social Support, Age, Educational Level, Number of Children and Marital Status

Masculine/instrumental and feminine/expressive traits were assessed by using The Bem Sex Role Inventory (BSRI) [62]. The BSRI is a self-report inventory that assesses participants’ identification with socially desirable personality traits that are stereotypically associated with women and men. The BSRI contains 60 items consisting of adjectives or short sentences, 20 of which refer to characteristics and traits traditionally regarded as masculine, such as “independent”, “competitive”, “dominant”, which make up the masculine/instrumental scale; 20 characteristics traditionally regarded as feminine, such as “compassionate”, “warm”, “gentle”, which make up the feminine/expressive scale; and 20 items formed by characteristics attributable to both genders, which were not used in the analysis. The response format is a 7-point Likert scale ranging from 1 (never or almost never true) to 7 (always or almost always true). The minimum possible value, both in the masculine/instrumental and feminine/expressive scale, was 20 and the maximum 140, and higher scores indicated greater self-identification with the characteristics of the trait. All items were translated into Spanish and back into English by the research team plus two bilingual persons, a native English-speaking professional translator, and a native speaker of Spanish. For the current sample, the Cronbach’s alpha in the men’s group of the 20 items on the masculine/instrumental scale was 0.84, and of the 20 items on the feminine/expressive scale was 0.85. In the women’s group, Cronbach’s alpha on the masculine/instrumental scale was 0.83, and on the feminine/expressive scale it was 0.85.

Social support was assessed by using the Social Support Scale [63]. It consisted of 12 items which measure the availability of social support. For each item, respondents are asked to rate on a 4-point Likert scale ranging from 0 (never) to 3 (always) the possibilities of access to other persons who can support and/or help with affective, economic, labor, familiar and advice/guidance needs. Confirmatory factor analyses, with a sample of 3210 people [64], showed that the 12 items were grouped into a factor in the women sample and in the total sample measuring global social support perceived. The minimum possible value was 0 and the maximum 36, and higher scores indicated greater social support perceived. The Cronbach’s alpha for the current sample was 0.91 in the men’s group and 0.92 in the women’s group.

Demographic variables included in the analysis were: participants’ age and number of children, that were treated as continuous variables; educational level, that was approached as an ordinal variable with seven levels, from 1 (for unfinished elementary studies) to 7 (for 5-year university degree); and marital status, that was included in regression analyses as a dummy variable with two levels: one included the never married, separated, divorced, and widowed (reference category, which was coded with 0) and in the other the married or living with a partner, which was coded with 1.

### 2.3. Procedure

This study forms part of an extensive research on gender and health and was positively evaluated by the Ethics Committee on Animal Research and Well-Being of the University of La Laguna (study approval number 2015-0170). The participants in the study were volunteers, and were not remunerated for their participation. To avoid systematic biases, access to the participants was through several retirees’ association centers of different Spanish localities, as well as by resorting to the social network (usually family and neighbors) of a large number of psychology, nursing, and sociology students from seven Spanish universities trained in administering those tests, who received course credits for that task.

After reported consent was obtained, tests were filled out individually, in some cases self-administered; in the case of participants with low educational level, or those who preferred to be interviewed, the tests were completed during the course of a structured interview carried out by trained university students. There were no significant differences across gender in the method of administering the tests. Test were answered manually on a paper version of the measures. Participants had to fulfill the following requirements: (1) age of 60 or more years; (2) not having cognitive or language problems that limited comprehension of the tests; (3) similar ages in women and men. No names or any other data identifying the participant were used in the tests. We have complied with American Psychological Association ethical standards in the treatment of the sample.

### 2.4. Statistical Analysis

Descriptive statistics were computed to describe the demographic characteristics of the participants. Comparisons between men and women were computed performing Pearson’s chi square test in case of categorical variables and by using *t*-test when they were continuous. The effect size of the mean differences was computed by using the Cohen’s *d*. The internal consistency reliability for the masculine/instrumental and feminine/expressive traits, social support and life satisfaction were calculated using the Cronbach’s alpha coefficient. Bivariate associations between variables were computed by using the correlation coefficient *r* of Pearson when they were quantitative variables and Spearman’s *Rho* when they were ordinal variables. Hierarchical multiple regression analyses were conducted to determine the relevance of the sociodemographic variables, the masculine/instrumental and feminine/expressive traits, and the social support in the self-rated health and men’s and women’s life satisfaction. The criterion considered was the score in self-rated health in the first regression analyses and the score in life satisfaction in the second. Age, educational level, number of children and marital status (as a dummy variable) were included in step 1. At step 2, masculinity-instrumental and feminine/expressive scores were entered. In step 3, social support was included. Statistical analyses were carried out using the software IBM SPSS Statistics for Windows, version 22.0 (IBM Corp., Armonk, NY, USA).

## 3. Results

### 3.1. Gender Differences in Life Satisfaction, Social Support, Masculine/Instrumental Trait, Feminine/ Expressive Trait, and Self-Rated Health

The 0.6% of men rated their health as very bad, 5.6% as bad, 40.3% as moderate, 42.9% as good and 10.7% as very good. The percentages in women were, respectively, 0.8%, 6.6%, 45.5%, 37.9% and 9.2%. The differences between the percentages were not statistically significant, χ^2^ (4, *N* = 1456) = 6.30, *p* = 0.178. Table 2 show the means, standard deviations and comparisons for men and women in life satisfaction, social support and masculine/instrumental and feminine/expressive traits. Statistically significant differences were found in three of the four variables. Men scored higher than women in masculine/instrumental trait and in life satisfaction, although the effect size for life satisfaction was small. And women scored higher than men in the feminine/expressive trait.

### 3.2. Predictors of Women’s and Men’s Self-Rated Health

Table 3 presents the correlation coefficients between dependent and independent variables calculated independently in the women and the men groups. In both genders, most of the correlation coefficients were statistically significant, although the magnitude of the association was not high. In addition, there were some differences in the correlations in the group of women and in the group of men, which highlighted that there were only statistically significant correlations between educational level and life satisfaction in the women group and between the feminine/expressive trait and self-rated health in the men group. In the latter, the number of children was associated with self-rated health.

Table 4 presents the standardized regression coefficients (*β*) with their corresponding *t* values, and the *F* and *R*^2^ values for the three regression models with the self-rated health as the dependent variable for the men group, and Table 5 for the women group.

Results identified that R for regression was significantly different from zero at the end of each step in both men and women groups. The sociodemographic variables entered into step 1 explained 4% of the variance in self-rated health in the men group and 6% in the women group. For men and women too age was statistically significant; in the men group, the number of children was also statistically significant whereas in the women group the statistically significant variable was the educational level. The change in *R*^2^ from model 1 to model 2 identified that the masculine/instrumental trait (*β* = 0.08, *p* < 0.05) and the feminine/expressive trait (*β* = 0.14, *p* < 0.001) played significant roles in men’s self-rated health, whereas in the women group it was the masculine/instrumental trait (*β* = 0.20, *p* < 0.001) that proved statistically significant. The addition of social support in Model 3 also yielded a statistically significant increment in *R*^2^ in the women and the men groups.

Beta values in Model 3, with all independent variables in the equation, showed that in the men group the significant predictors of self-rated health were social support, age, feminine/expressive trait, and number of children, so men with more social support, less age, higher feminine/expressive trait and lower number of children reported better self-rated health. In the women group, the significant predictors were social support, educational level, masculine/instrumental trait, and age. Women with more social support, higher educational level and masculine/instrumental trait and less age reported better self-rated health.

### 3.3. Predictors of Women’s and Men’s Life Satisfaction

Table 6 provides the main results of the hierarchical regression with life satisfaction as the dependent variable for the men group, whereas results from Table 7 correspond to the women group.

*R* for regression was significantly different from zero at the end of each step in the women group and only at the end of steps 2 and 3 in the men group. In the women group, after model 1, with age, educational level, number of children and marital status in the equation, *R*^2^ = 0.04, *p* < 0.001. In both genders, the change in *R*^2^ from model 1 to model 2 identified that masculine/instrumental and feminine/expressive traits determine significantly men’s and women’s life satisfaction. The addition of social support in model 3 resulted in a statistically significant (*p* < 0.001) increment in *R*^2^ (*R*^2^ change = 0.13 in the men group and *R*^2^ change = 0.12 in the women group). Beta values in the model 3, with all independent variables in the equation, proved that social support was the variable most associated with men’s and women’s life satisfaction; the second most relevant variable was masculine/instrumental trait. Another statistically significant predictor for both genders was age. Life satisfaction was higher in women and men with high social support, high masculine/instrumental trait and more age. In addition, in the case of women, educational level, marital status, and feminine/expressive trait were also significant predictors. Thus, life satisfaction scored higher in women with higher educational level, high social support, older age, married or cohabiting marital status and with a high score in both the masculine/instrumental and the feminine/expressive trait.

## 4. Discussion

Although in recent decades the social determinants of health have received considerable attention in the field of public health [60], few studies had focused on the elderly and fewer had approached the relevance of gender for successful ageing [65,66]. Although there is a growing social awareness of the phenomenon called ‘feminization of old age’ “the topic and its psychosocial and health-related consequences have not attracted the scientific interest that they deserve” ([65], p. 208). The main aim of this work was to analyze the relevance that two important social determinants of health, gender and social support, have on self-rated health and life satisfaction in elderly Spanish people. Spain is one of the countries in the world with the highest life expectancy at birth and, together with Japan, they lead a group of 25 Organization of Economic Co-operation and Development (OECD) countries with life expectancy over 80 years [67]. As in most countries, life expectancy in Spain is greater in women than in men, with life expectancy at birth of 80.3 years in men and 85.7 in women [26]. Although it is an important achievement, the aging of the population poses important challenges, since it implies greater economic and social needs [68].

The first hypothesis of the study predicting that men would have better self-rated health than women was not supported. Although research has reported that women’s self-rated health was worse than men’s [36,37], gender gaps vary cross-nationally [38,67]. Findings did also reveal that the differences between women and men in self-rated health vary in agreement with other behavioral and demographic variables [30,37] and that female disadvantage disappears when socio-economic and health covariates are considered [69]. Although findings in the present study have provided no statistically significant gender differences in self-rated health, it may be owing to the fact that this research has been done with people who participated voluntarily, so access to part of the sample was through retirees’ association centers; therefore, there may have been a certain bias towards greater participation of people with better health, which may be more common in women than in men. In fact, very few people rated their health as very bad. Older people’s self-rated health has been also associated with demographic variables, as ill health reporting increases with age [3,67]; these results are consistent with those provided by this study where worse self-rated health has been found at older ages, although the strength of the association was greater in men than in women.

The second hypothesis predicting that women and men with a higher educational level would have better self-rated health and greater life satisfaction was only supported in the women sample. In the correlation analysis between dependent and independent variables for the men group, it was found that, although educational level correlated statistically significantly with self-rated health, the magnitude of the association was very low (*r* = 0.11). In the hierarchical regression analyses the Beta weights for the educational level were not statistically significant in the group of men in any of the three models, which was observed either when the dependent variable was self-reported health or when it was life satisfaction. Although the positive association between education and health and survival is well established, gender could be an important variable in such association [70]. Ross et al. [70] found that education’s beneficial influence on health and on survival were determined by gender but in the opposite ways, since education has a larger effect on women’s self-rated health than on men’s, but a larger effect on men’s mortality due to causes such as lung cancer, stroke, respiratory disease, suicide, homicide, and accidents.

The third hypothesis, predicting that women and men with higher scores on the masculine/instrumental trait would report better self-rated health and greater life satisfaction, was not fully supported. Although women and men scoring higher in the masculine/instrumental trait had higher life satisfaction, in the final model, when social support was introduced, the association between the masculine/instrumental trait and self-rated health only occurred in the case of women. The gender trait that predicted self-rated health in men was the feminine/expressive, with better health in men who scored higher; and the feminine/expressive trait proved to be independent of women’s health. These results underline the relevance of identifying with the characteristics stereotypically associated with gender when addressing health and well-being in the elderly; self-rated health is better in women and men whose self-concept includes the characteristics of the other gender, that is, the masculine/instrumental trait in the case of women and the feminine/expressive in that of men. The underlying reason may be that women who score higher in masculine/instrumental trait are better able to defend their rights and to dedicate more time to themselves and their self-care; whereas men who score higher in feminine/expressive trait are better able to ask for help if case they need, and take better care of their health. It also makes clear how the exclusive identification with the characteristics associated with the same gender, such as independence, dominance, self-sufficiency, or individualism in the case of men, and sensitivity to the needs of others, sympathy, kindness, or warmth in the case of women, is a limitation for health and well-being. The results provided converge and support the literature that states that openness to positive traits of the opposite gender role is an essential trait for successful ageing [66]. Moreover, the research conducted with the general population has found that people whose self-concept includes both instrumental and expressive characteristics report greater self-rated health and well-being [19,22,23,71]. These results could be important for the design of interventions with the elderly, which should include strategies for women to develop characteristics such as independence, self-confidence, individuality, and assertiveness; and for men to develop empathy, sensitivity to the needs of others, or warmth.

The fourth hypothesis predicting that women and men with higher scores on social support would have better self-rated health and greater life satisfaction was supported. These results converge and extend the existing literature regarding the relevance of social support in the elderly’s life satisfaction [55,72,73].

The results of this work show the relevance of gender in the elderly’s health and well-being, although in both genders a high social support is associated with better self-rated health and higher life satisfaction. Age was also relevant in both men and women but its effect was different depending on the indicator of health in question: older age was associated with lower self-rated health—which was greater in the case of men than in women—but with greater life satisfaction. Research carried out in several countries on the relationship between age and life satisfaction does not show consistent results [59], having found that this relationship depends on other variables such as the age group or the country where the study was carried out [59,74]. Literature for most European countries and the USA documents a U-shaped relationship between both variables (life satisfaction decreasing to midlife, to increase subsequently towards retirement), although the literature that claims to find a U shape of life satisfaction over age has been criticized [75]. Although there are important individual differences, it has been found that life satisfaction does not decrease, but rather increases from 40 to 65 years “before declining only close to impending death” ([59], p. 397). Diener et al. [59] affirm that, although it is necessary to increase research in this area, “it is clear that old age is not necessarily a harbinger of unhappiness” (p. 397). The results of the present study on the association between age and life satisfaction converge with this affirmation and expand the empirical evidence that sustains it.

In the present study it has also been found that, while a high educational level is associated with better self-rated health and higher life satisfaction in the women group, this is not the case for the men group. In addition, being married or cohabiting was associated with greater life satisfaction only in the women group. Although the reasons for this are unknown, in interpreting these results it should be taken into account that, in the present sample, there were differences between women and men as for the educational level, which was higher in men than in women, and for the marital status, since being married of cohabiting was more common in men than in women, whereas the percentage of widows was higher than that of widowers. Although the sample of the present study is not random, so that results cannot be generalized to the general population, it is important to bear in mind that these differences between women and men are coherent with the composition of the Spanish population of elderly people, which presents a higher rate of women with lower levels of education than men, and a higher occurrence of widows [76].

Although the results of the present work allow to advance in the knowledge of the social determinants of health and well-being of the elderly, they also present some limitations. The first one is that this is a cross-sectional study, therefore, no cause-effect inferences can be made. Second, the sample, though large and with women and men with different demographic characteristics, is a convenience sample. Third, all participants lived in Spain, which may restrict the generalization of results with respect to other countries. Fourth, the percentage of the variance explained in self-rated health by social support, masculine/instrumental trait, age, and educational level in the case of women, and by social support, age, feminine/expressive trait and number of children in men, was low. Fifth, the measures were administered to some participants by interviewers, in the form of a structured interview, which could have biased some responses. Future research is needed to investigate the causal link between the variables, to assess the generalizability of these results in other countries, as well as to expand knowledge of the variables that determine the self-rated health of elderly women and men.

## 5. Conclusions

Gender and social support are important social determinants of health among the elderly, as people having greater social support present better self-rated health and greater life satisfaction. In addition, women with higher education have better self-rated health and higher life satisfaction. Identification with traits associated with traditional gender roles has been shown to be an important factor in self-rated health and life satisfaction, and women and men whose self-concept includes both instrumental and expressive characteristics are more likely to have greater health and wellbeing. Against gender stereotypes that characterize women and men as complementary and that prescribe that men should be agentic, independent and instrumental but not communal, warm or expressive, and that women should be communal, warm or expressive but not agentic, independent and instrumental, the results of this work show that both types of characteristics are associated with the health and well-being of both women and men. The results of this study can be useful for the design of policies, programs and strategies aimed at a more successful aging of women and men and for the increase of gender equality.

## Figures and Tables

**Table 1:** Demographic characteristics of the men and women groups.

	Men (*n* = 702)		Women (*n* = 754)		χ^2^-Value
	*n*	%	*n*	%	
Education					
Unfinished elementary studies	98	14.0	126	16.7	46.23 ***
Elementary studies	256	36.5	335	44.4	
First grade professional training	33	4.7	41	5.4	
High school degree	145	20.7	97	12.9	
Second grade professional training	23	3.3	19	2.5	
3-year university degree	60	8.5	94	12.5	
5-year university degree	87	12.4	42	5.6	
Marital Status					
Never married	38	5.4	51	6.8	96.85 ***
Married/cohabiting	550	78.3	429	56.9	
Separated/divorced	51	7.3	70	9.3	
Widowed	63	9.0	204	27.1	
	***M***	***SD***	***M***	***SD***	***t*-Value**
Age	69.79	6.48	70.17	6.55	−1.10
Number of children	2.42	1.52	2.58	1.60	−1.94

Note: *** *p* < 0.001.

**Table 2:** Means (*M*), standard deviations (*SD*) and comparisons for men and women for the quantitative study variables.

	Men (*n* = 702)		Women (*n* = 754)		*t*_(1454)_	*d*-Value
	M	SD	M	SD		
Life satisfaction	24.79	6.08	23.83	6.84	2.85 **	0.15
Social support	25.47	7.73	26.10	7.81	−1.57	−0.08
Masculine/instrumental trait	94.80	15.46	83.47	15.89	13.77 ***	0.72
Feminine/expressive trait	92.09	14.05	102.04	13.77	−13.64 ***	−0.71

Note: ** *p* < 0.01; *** *p* < 0.001; *d*-value = Cohen’s *d*.

**Table 3:** Correlations between dependent and independent variables for men and women groups.

	Men		Women	
	Life Satisfaction	Self-Rated Health ^$^	Life Satisfaction	Self-Rated Health ^$^
Age	0.08 *	−0.18 ***	0.04	−0.14 ***
Educational level ^$^	0.03	0.11 **	0.13 ***	0.25 ***
Number of children	0.07	−0.13 **	−0.06	−0.06
Masculine/instrumental trait	0.30 ***	0.12 **	0.27 ***	0.23 ***
Feminine/expressive trait	0.20 ***	0.20 ***	0.21 ***	0.01
Social support	0.43 ***	0.17 ***	0.42 ***	0.21 ***

Note: * *p* < 0.05; ** *p* < 0.01; *** *p* < 0.001; ^$^ = Spearman’s *Rho* correlation coefficient.

**Table 4:** Summary of the hierarchical regression with self-rated health as the dependent variable for the men group.

	Model 1		Model 2		Model 3	
	*β*	*t*-Value	*β*	*t*-Value	*β*	*t*-Value
Age	−0.15	−3.62 ***	−0.13	−3.12 **	−0.14	−3.45 ***
Educational level	0.05	1.17	0.03	0.75	0.03	0.68
Number of children	−0.09	−2.11 *	−0.09	−2.14 *	−0.09	−2.19 *
Married/cohabiting	0.01	0.20	0.00	0.07	−0.02	−0.59
Masculine/instrumental trait			0.08	2.20 *	0.07	1.82
Feminine/expressive trait			0.14	3.79 ***	0.11	2.96 **
Social support					0.14	3.58 ***
*R* ^2^	0.05		0.08		0.09	
Adjusted *R*^2^	0.04		0.07		0.09	
*R*^2^ Change	0.05		0.03		0.02	
*F* Change	8.19 ***		12.15 ***		12.84 ***	
ANOVA (*F*-value, df)	8.19 (4, 697) ***		9.68 (6, 695) ***		10.28 (7, 694) ***	

Note: *β* = Standardized regression coefficient; *R*^2^ = percentage of explained variance; * *p* < 0.05; ** *p* < 0.01; *** *p* < 0.001.

**Table 5:** Summary of the hierarchical regression with self-rated health as the dependent variable for the women group.

	Model 1		Model 2		Model 3	
	*β*	*t*-Value	*β*	*t*-Value	*β*	*t*-Value
Age	−0.08	−2.01 *	−0.05	−1.38	−0.08	−2.03 *
Educational level	0.22	5.76 ***	0.18	4.71 ***	0.18	4.78 ***
Number of children	−0.01	−0.19	−0.00	−0.10	−0.01	−0.36
Married/cohabiting	0.02	0.50	0.03	0.69	0.01	0.25
Masculine/instrumental trait			0.20	5.41 ***	0.17	4.69 ***
Feminine/expressive trait			−0.00	−0.07	−0.03	−0.85
Social support					0.19	5.23 ***
*R* ^2^	0.07		0.11		0.14	
Adjusted *R*^2^	0.06		0.10		0.13	
*R*^2^ Change	0.07		0.04		0.03	
*F* Change	13.49 ***		15.67 ***		27.32 ***	
ANOVA (*F*-value, df)	13.49 (4, 749) ***		14.57 (6, 747) ***		16.83 (7, 746) ***	

Note: *β* = Standardized regression coefficient; *R*^2^ = percentage of explained variance; * *p* < 0.05; *** *p* < 0.001.

**Table 6:** Summary of the hierarchical regression with life satisfaction as the dependent variable for the men group.

	Model 1		Model 2		Model 3	
	*β*	*t*-Value	*β*	*t*-Value	*β*	*t*-Value
Age	0.08	1.89	0.11	2.90 **	0.08	2.18 *
Educational level	0.05	1.29	0.01	0.23	0.00	0.01
Number of children	0.04	0.97	0.03	0.68	0.02	0.64
Married/cohabiting	0.05	1.26	0.04	1.16	−0.03	−0.75
Masculine/instrumental trait			0.27	7.33 ***	0.23	6.72 ***
Feminine/expressive trait			0.15	3.94 ***	0.06	1.81
Social support					0.38	10.85 ***
*R* ^2^	0.01		0.12		0.25	
Adjusted *R*^2^	0.01		0.12		0.24	
*R*^2^ Change	0.01		0.11		0.13	
*F* Change	2.33		43.55 ***		117.68 ***	
ANOVA (*F*-value, df)	2.33 (4, 697)		16.26 (6, 695) ***		33.09 (7, 694) ***	

Note: *β* = Standardized regression coefficient; *R*^2^ = percentage of explained variance; * *p* < 0.05; ** *p* < 0.01; *** *p* < 0.001.

**Table 7:** Summary of the hierarchical regression with life satisfaction as the dependent variable for the women group.

	Model 1		Model 2		Model 3	
	*β*	*t*-Value	*β*	*t*-Value	*β*	*t*-Value
Age	0.13	3.27	0.14	3.77 ***	0.10	2.70 **
Educational level	0.17	4.32	0.12	3.27 **	0.12	3.49 **
Number of children	−0.05	−1.36	−0.04	−1.22	−0.06	−1.85
Married/cohabiting	0.11	3.08	0.12	3.29 **	0.09	2.59 *
Masculine/instrumental trait			0.23	6.26 ***	0.18	5.09 ***
Feminine/expressive trait			0.14	3.79 ***	0.08	2.45 *
Social support					0.36	10.76 ***
*R* ^2^	0.04		0.13		0.24	
Adjusted *R*^2^	0.04		0.12		0.24	
*R*^2^ Change	0.04		0.08		0.12	
*F* Change	8.44 ***		35.68 ***		115.84 ***	
ANOVA (*F*-value, df)	8.44 (4, 749) ***		18.04 (6, 747) ***		34.39 (7, 746) ***	

Note: *β* = Standardized regression coefficient; *R*^2^ = percentage of explained variance; * *p* < 0.05; ** *p* < 0.01; *** *p* < 0.001.
